# The multifaceted roles of mesenchymal stem cell-derived exosomes in digestive system malignancies: mechanisms and therapeutic implications

**DOI:** 10.3389/fcell.2026.1814232

**Published:** 2026-04-20

**Authors:** Boyu Li, Ziheng Cui, Yanhua Jin

**Affiliations:** Department of Cell Biology and Medical Genetics, College of Medicine, Yanbian University, Yanji, Jilin, China

**Keywords:** cancer, digestive system tumors, exosomes, mesenchymal stem cell-derived exosomes, mesenchymal stem cells

## Abstract

Mesenchymal stem cell-derived exosomes (MSC-Exos) have emerged as key mediators of intercellular communication within the tumor microenvironment. However, a comprehensive synthesis of their paradoxical roles in digestive system tumors remains absent. This review provides an in-depth analysis of the molecular mechanisms by which MSC-Exos regulate tumor progression, with a focus on how they transfer specific non-coding RNAs and proteins to target cells, thereby modulating angiogenesis, epithelial-mesenchymal transition, immune evasion, and drug resistance. We highlight the functional heterogeneity of MSC-Exos in colorectal, liver, gastric, and pancreatic cancers, and examine how signals from the tumor microenvironment remodel their molecular cargo, establishing complex feedback loops. Furthermore, we discuss emerging translational frontiers, including the engineering of MSC-Exos as targeted drug delivery vehicles. By integrating mechanistic insights with clinical challenges, this review aims to elucidate the complex biology of MSC-Exos and pave new avenues for their application in precision oncology for digestive system tumors.

## 1 Introduction

Digestive system tumors represent one of the most prevalent malignancies worldwide and a leading cause of cancer-related mortality. In China, the burden of digestive system tumors is particularly substantial. With accelerating population aging and shifting lifestyles, incidence rates continue to rise, projected to reach 2.92 million cases by 2050—a 49.06% increase from 2021 (Lu et al., 2025). This escalating burden poses significant challenges to China’s healthcare system. Despite advances in medical research, the diagnosis and treatment of digestive system tumors face multiple challenges: low early detection rates, significant adverse effects of conventional therapies, and frequent drug resistance. These factors collectively contribute to poor patient prognosis. Therefore, novel and effective therapeutic strategies are urgently needed to improve patient outcomes.

Exosomes hold substantial promise in cancer research and therapy. On one hand, the distinctive molecular content of exosomes makes them valuable as biomarkers for early cancer diagnosis (Wang F. et al., 2025). On the other hand, exosomes play a critical role in tumorigenesis and progression by modulating processes such as cell proliferation, differentiation, and immune responses. Recent studies further highlight the potential of exosomes in mediating chemotherapy resistance and facilitating targeted drug delivery, offering innovative strategies for cancer treatment (Lin et al., 2022). This review focuses on mesenchymal stem cell-derived exosomes (MSC-exosomes), reviewing their research advancements in common digestive system tumors. It aims to provide a solid theoretical foundation and practical insights for clinical applications, thereby contributing to the advancement of cancer treatment.

## 2 Literature retrieval and screening methods

### 2.1 Search conductor and timeframe

The literature search was independently conducted by the first author in January 2026.

### 2.2 Publication types

The included literature encompasses original research articles, systematic reviews, narrative reviews, clinical experience exchanges, and meta-analyses.

### 2.3 Inclusion and exclusion criteria

Inclusion criteria were: (1) studies investigating mesenchymal stem cell-derived exosomes in the context of digestive system tumors; and (2) research literature pertaining to mesenchymal stem cell-derived exosomes and cancer biology. Exclusion criteria comprised: (1) publications with limited contemporary relevance due to extended time since publication; and (2) non-English and non-Chinese publications.

### 2.4 Search strategy

A computerized search was performed across multiple databases, including PubMed and Web of Science. A combination of Medical Subject Headings (MeSH) terms and keywords was employed. The search terms included: “mesenchymal stem cells”, “exosomes”, “mesenchymal stem cell derived exosomes”, “cancer”, “colorectal cancer”, “liver cancer”, “gastric cancer”, “pancreatic cancer”, “esophageal cancer”, and “cholangiocarcinoma.” The search timeframe encompassed literature from the inception of each database to January 2026.

## 3 MSC-exos overview

**FIGURE 1:**
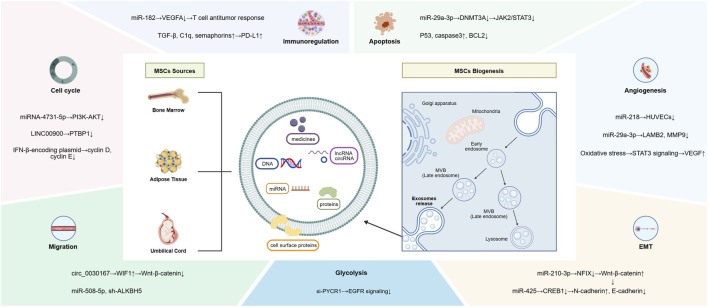
Schematic diagram of exosomes biogenesis and composition. Created with BIOGDP.com (Jiang S. et al., 2025).

Exosomes represent a distinct class of extracellular vesicles, typically ranging from 40 nm to 200 nm in diameter, that are generated through the endosomal pathway. Specifically, they are formed as intraluminal vesicles within multivesicular bodies and are subsequently released into the extracellular space upon fusion of the multivesicular body with the plasma membrane. Exosomes can transport a diverse array of biological macromolecules, including proteins, lipids, and nucleic acids. Through specific interactions with target cells, exosomes facilitate the transport of their contents across cellular membranes and mediate intercellular information exchange (Krylova and Feng, 2023) (Figure 1). Since their discovery in sheep reticulocytes in 1983, exosomes have been shown to originate from various cell types and exhibit diverse functions, with their composition displaying significant heterogeneity. Mesenchymal stem cells (MSCs) are versatile stem cells known for their strong regenerative capacity and the potential to differentiate into multiple cell types. They are widely recognized as efficient producers of exosomes (Yeo et al., 2013). Mesenchymal stem cell-derived exosomes have emerged as a promising nanoparticle platform for targeted cancer therapy. Compared to synthetic nanoparticles, these natural membrane vesicles offer advantages including high biocompatibility, low immunogenicity, minimal cytotoxicity, good stability, and enhanced tumor cell internalization. (Weng et al., 2021). MSC-Exos themselves exhibit tumor-suppressive activity in various cancer types and can be engineered to deliver therapeutic agents via extrusion, electroporation, dialysis, or saponin-assisted encapsulation (Raghav and Mann, 2024). Notably, MSC-derived microparticles retain membrane chemokine receptors that enable specific tumor homing while maintaining minimal toxicity toward normal cells. This targeting capability positions MSC-Exos as an ideal platform for precise delivery of therapeutic cargo to cancer stem cells, holding great potential for eradicating the CSC population and preventing tumor recurrence (Raghav and Mann, 2021).

## 4 The mechanism of MSC-Exo in tumor

### 4.1 Mechanisms governing exosomal cargo sorting and functional reprogramming

MSC-Exos display functional duality in tumors, functioning either as suppressors or promoters of cancer progression. This plasticity is largely determined by selective cargo packaging through distinct molecular mechanisms, as opposed to passive encapsulation of cellular contents. Sorting is dynamically regulated by cellular origin, microenvironmental cues, and specific trafficking pathways, which collectively define exosomal composition and functional output.

#### 4.1.1 ESCRT-dependent and independent pathways

Exosome biogenesis involves both endosomal sorting complexes required for transport (ESCRT)-dependent and ESCRT-independent mechanisms (Hessvik and Llorente, 2018). The ESCRT machinery recognizes ubiquitinated proteins and directs their incorporation into intraluminal vesicles of multivesicular bodies; disruption of key ESCRT components substantially alters exosomal cargo profiles and biological activity (Colombo et al., 2013). In parallel, tetraspanins CD9, CD63, and CD81 organize into membrane microdomains that facilitate the enrichment of non-ubiquitinated proteins and specific lipids, contributing to molecular diversity (Villarroya-Beltri et al., 2013).

#### 4.1.2 Mechanisms underlying selective miRNA packaging

Selective incorporation of non-coding RNAs, including microRNAs (miRNAs), relies on defined sequence motifs and recognition by RNA-binding proteins (RBPs). Proteins such as hnRNPA2B1 and SYNCRIP bind EXO-motifs within miRNAs, mediating their targeted enrichment into exosomes (Zhang et al., 2015). This mechanism ensures context-dependent packaging of functionally relevant miRNAs, including miR-210 and miR-652–3p, under specific physiological or pathological conditions.

#### 4.1.3 Microenvironment-driven functional reprogramming

Exosomal cargo sorting is dynamically influenced by microenvironmental signals. Under hypoxic conditions, hypoxia-inducible factor 1-alpha (HIF-1α) activation upregulates miR-210 and promotes its exosomal enrichment (Li et al., 2023); concurrently, hypoxia-induced miR-652–3p targets TNRC6A, attenuating tumor-suppressive functions (Almouh et al., 2024). Oxidative stress remodels the immunomodulatory cargo of MSC-Exos via STAT3 signaling, thereby enhancing angiogenesis and tumor progression (Kalluri and LeBleu, 2020). Collectively, these microenvironmental factors modulate the activity of sorting machinery, driving the functional switch of MSC-Exos from tumor inhibition to tumor promotion.

### 4.2 Effect of MSC-Exos on tumor growth

Following treatment with umbilical cord-derived mesenchymal stem cells (U-MSC) and their derived exosomes (U-MSC-Exos), the effects on tumor cell proliferation exhibited tissue-specific patterns. Proliferation was significantly enhanced in lung cancer A549 cells, breast cancer MDA-MB-231 cells, and gastric cancer BGC-823 cells, whereas it was diminished in glioma LN-229 cells (Liu et al., 2023). This observation confirms that the functional impact of exosomes depends not only on their cellular origin and molecular cargo but also on the recipient tumor type and its intrinsic signaling networks. From a mechanistic perspective, exosomes can effectively penetrate the dense extracellular matrix and hypoxic regions within the tumor microenvironment by virtue of their nanoscale size, flexible lipid bilayer, and surface-specific receptors, thereby enabling efficient targeted delivery of bioactive components. However, heterogeneity among recipient tumor cells—including differences in metabolic state, membrane protein expression, and endocytic efficiency—directly influences exosome uptake kinetics and intracellular delivery efficiency, ultimately modulating their effects on cell proliferation and other phenotypes (Zhang Q. et al., 2025). With respect to inhibitory regulation, U-MSC-Exos overexpressing miR-503–3p suppress endometrial cancer cell growth (Pan et al., 2022). Adipose-derived mesenchymal stem cell exosomes (AD-MSC-Exos) transfected with miR-4731–5p target and regulate the phosphatidylinositol 3-kinase/protein kinase B (PI3K/Akt) and nuclear factor kappa-B (NF-κB) signaling pathways, significantly increasing the proportion of U-251 and U-87 glioma cells in G0/G1 phase while reducing S-phase populations, thereby inducing cell cycle arrest (Babaei et al., 2024). Similarly, the long non-coding RNA linc00900 delivered by AD-MSC-Exos inhibits polypyrimidine tract-binding protein 1 (PTBP1), suppressing autophagy, inducing G0/G1 arrest, and promoting apoptosis. This mechanism significantly inhibits thyroid cancer cell proliferation and tumor growth both *in vitro* and *in vivo* (An et al., 2026). With advancing research, precision regulation strategies based on gene editing technologies have demonstrated potential for clinical translation. Inhibiting pyrroline-5-carboxylate reductase 1 (PYCR1) expression in bone marrow-derived MSC-Exos (B-MSC-Exos) modulates the epidermal growth factor receptor (EGFR)-mediated PI3K/Akt signaling pathway, suppressing aerobic glycolysis and tumor cell proliferation. Building on this finding, nano-nucleic acid drugs utilizing MSC-Exos as delivery vehicles can effectively inhibit tumor progression (Li et al., 2023). Collectively, these findings demonstrate that through gene editing or drug loading technologies, the function of MSC-Exos can be transformed from an “environment-dependent” carrier to a “function-controllable” therapeutic tool (Figure 1).

### 4.3 Effect of MSC-Exos on tumor immune microenvironment

The regulation of the tumor immune microenvironment by MSC-Exos exhibits considerable duality and context dependence, rendering them both a promising tool and a significant challenge in tumor immunotherapy. The net effect is largely determined by the immunomodulatory molecules carried by exosomes and the local microenvironmental signals. In terms of immune activation, MSC-Exos can directly or indirectly enhance anti-tumor immune responses by delivering specific miRNAs (Figure 1). For instance, U-MSC-Exos not only increase the numbers of dendritic cells (DCs), natural killer T cells, and CD8^+^ T cells in murine spleen and tumor tissues, but also promote T cell proliferation and maturation *in vitro*. Furthermore, they potentiate T cell-mediated immune responses by delivering miR-182, which targets vascular endothelial growth factor A (VEGFA) expression, thereby alleviating cancer progression (Li D. et al., 2021). Conversely, within the specific tumor microenvironment, MSC-Exos may also exert immunosuppressive effects that facilitate immune evasion. For example, in breast cancer models, MSC-Exos drive monocytic myeloid-derived suppressor cells (MDSCs) to differentiate into M2 tumor-associated macrophages (TAMs) by carrying immunomodulatory molecules such as transforming growth factor-beta (TGF-β), complement C1q, and semaphorins. These M2 TAMs highly express programmed death-ligand 1 (PD-L1) and exhibit enhanced L-arginase activity and interleukin-10 (IL-10) secretion. Collectively, these changes significantly inhibit T cell activity within the tumor microenvironment, promote tumor immune escape, and accelerate disease progression (Biswas et al., 2019). Therefore, future research should further clarify the key molecular and microenvironment factors that determine the direction of its immune regulation, so as to play an anti-tumor role.

### 4.4 Effect of MSC-Exos on tumor angiogenesis

Tumor angiogenesis is a critical step in tumor progression and metastasis, and MSC-Exos exert a complex, bidirectional influence on this process (Figure 1). The heterogeneous origins and molecular cargo of exosomes constitute the fundamental basis for their functional diversity. Studies have shown that miR-29a-3p in bone marrow-derived mesenchymal stem cell exosomes (B-MSC-Exos) targets the Roundabout guidance receptor 1 (Robo1) gene to inhibit angiogenesis in glioma (Zhang et al., 2021). This contrasts with the function of exosomes released by MSCs pretreated under specific stress conditions. For instance, the cargo composition of MSC-Exos derived from somatic tissues under oxidative stress is altered, leading to upregulation of vascular endothelial growth factor (VEGF) expression via activation of the signal transducer and activator of transcription 3 (STAT3) signaling pathway in breast cancer cells. This, in turn, promotes endothelial cell proliferation and angiogenesis by elevating reactive oxygen species levels (Almouh et al., 2024). Given the variability in MSC-Exo origin, molecular composition, and target cell types in tumor therapy, precisely regulating MSC-Exo function while minimizing potential adverse effects remains a critical priority for future research.

### 4.5 Effect of MSC-Exos on tumor metastasis

Tumor metastasis is a multi-step process and is the leading cause of cancer-related mortality. Consequently, inhibiting metastasis represents a critical therapeutic strategy in oncology research (Figure 1). Studies have shown that MSC-Exos from different sources can interfere with the key pathways of metastasis by delivering specific RNA molecules. B-MSC-Exos can inhibit the invasion, migration and proliferation of pancreatic cancer cells through miR-338–5p*/Wnt inhibitor factor 1 (Wif1)/Wnt family member 8 (wnt8)*/β-catenin axis (Yao et al., 2021). Overexpression of miR-508–5p in human MSCs through lentiviral transduction technology can restore the function of the miRNA in endometrial cancer cells, and then target to inhibit the oncogene *delta like ligand 3 (DLL3)*, which can significantly inhibit the proliferation, migration and metastasis of tumors (Li et al., 2025). In breast cancer, MSC-Exos can not only inhibit the stem cell characteristics and metastasis of triple negative breast cancer cells through the *alkB homolog 5*-dependent mechanism in B-MSC-Exos, but also inhibit the epithelial-mesenchymal transition (EMT) and angiogenesis of triple negative breast cancer cells and inhibit the invasion of cancer cells through AD- MSC-Exos rich in miR-218 (Hu et al., 2022; Shojaei et al., 2023). These results indicate that exosomes have the potential of multi-target and multi-channel regulation in anti-metastasis therapy. However, due to the heterogeneous composition of MSC-Exos, they can also promote tumor metastasis by delivering specific miRNAs. For example, B-MSC-Exos can deliver miR-425 to inhibit cytoplasmic polyadenylation element-binding protein 1 (CPEB1) expression in lung cancer cells, thereby enhancing their migration and invasion (Wang et al., 2022). Collectively, MSC-Exos exert bidirectional regulatory effects on tumor metastasis by modulating RNA delivery, protein expression, and cytokine secretion.

### 4.6 Effect of MSC-Exos on apoptosis of tumor cells

Apoptosis, a principal form of programmed cell death, plays a central role in tumor development and therapeutic response, representing a key endpoint for many anticancer strategies. Emerging evidence highlights the regulatory function of MSC-Exos in tumor cell apoptosis (Figure 1), with their effects being context-dependent—capable of either suppressing or promoting apoptosis. Under certain conditions, MSC-Exos may play an anti apoptotic role. For example, under hypoxic conditions, B-MSC-Exos can target inhibition of *nuclear factor IX* by delivering miR-210–3p, consequently activating Wnt/β - catenin signaling pathway. This promotes the proliferation, migration, invasion and EMT of triple negative breast cancer cells *in vitro* and *in vivo*, and inhibiting their apoptosis (Wang M. et al., 2025). This observation suggests that upon MSC recruitment into the tumor microenvironment and their aberrant interaction with it, the function of their exosomes may be altered to support tumor survival. However, in most solid tumors, MSC-Exos function as pro-apoptotic factors, inducing cell apoptosis and necrosis, inhibiting cell proliferation, and thereby exerting anticancer effects (Rezaeian et al., 2022). Studies have demonstrated that adipose-derived MSC-Exos induce apoptosis in bladder cancer 5637 cells and prostate cancer LNCaP and PC3 cells, as evidenced by increased TP53 expression, decreased BCL-2 expression, and an elevated proportion of Annexin V-positive cells, confirming that MSC-Exos promote apoptosis through regulation of the p53/BCL-2 axis. Notably, the apoptotic response to MSC-Exos varies across different tumor cells: in bladder cancer cells, downregulation of BAX and VEGFA was observed in addition to BCL-2 reduction, suggesting the existence of cell type-specific apoptosis regulatory networks (Rezaeian et al., 2022). In non-small cell lung cancer, B-MSC-Exos inhibit proliferation and migration while promoting apoptosis of cancer cells by delivering miR-29a-3p, specifically inhibiting *DNA methyltransferase 3a* and down regulating Janus kinase 2 signal transducer and activator of transcription 3 (JAK2/STAT3) signaling pathway (Su et al., 2025). Similarly, AD-MSC-Exos can induce the expression of pro-apoptotic genes and promote apoptosis in breast cancer cell lines (Felthaus et al., 2024). These studies show that the exosomes of stem cells without abnormal signal interference often play a role in promoting cell apoptosis. It is noteworthy that the pro apoptotic process can be actively strengthened and targeted through engineering transformation. Yuan et al. found that U-MSC-Exos modified by interferon - β (IFN-β) could induce G0/G1 phase arrest of prostate cells PC3 and LNCaP, significantly enhance its ability to induce prostate cancer cell cycle arrest and apoptosis, thereby inhibiting the proliferation of cancer cells and promoting apoptosis (Yuan et al., 2023).

In conclusion, MSC-Exos do not play a single role in tumor regulation, but as a dynamic information carrier (Figure 1). Future research should explore engineering strategies, such as loading immune agonists or targeted editing of anti-tumor related miRNA to enhance its anti-tumor function, so as to transform it into a stable and controllable anti-tumor tool.

## 5 Research progress of MSC-Exos in digestive system tumors

### 5.1 Effect of MSC-Exos on CRC

#### 5.1.1 The anti-cancer effect of MSC-Exos on CRC

CRC has become the second leading cause of cancer death in the world, and its treatment faces multiple challenges such as chemotherapy resistance and immunosuppressive microenvironment. Because of its natural biocompatibility and targeting, MSC-Exos shows important value in the treatment of CRC (Table 1). In terms of natural regulation, miR-486–5p in U-MSC-Exos can inhibit its expression by targeting the 3′-untranslated region of *NIMA-related kinase 2 (Nek2)* mRNA. This regulation not only inhibits the proliferation, migration and invasion of CRC cells, but also significantly inhibits the aerobic glycolysis and tumor stemness of CRC cells (Cui et al., 2024). The concurrent suppression of glycolysis and stemness by hUC-MSC-EXOs suggests a mechanistic link. Glycolysis supplies NADPH to counter oxidative stress; its inhibition may disrupt redox balance, altering ROS levels. Given that ROS modulate stemness in a concentration-dependent manner—promoting it at moderate levels while inducing cell death at high levels (Glorieux et al., 2024)—this underscores the need for precise modulation of glycolytic inhibition to achieve therapeutic benefit without unintended pro-tumor effects. This mechanism reveals that MSC-Exos can simultaneously interfere with the two core malignant phenotypes of tumor metabolism reprogramming and stemness by delivering specific miRNAs, which provides a new perspective for targeting CRC from the perspective of energy metabolism. Similarly, miR-1827 in U-MSC-Exos inhibits the energy metabolism, proliferation, metastasis and invasion of CRC cells by down regulating the expression of energy metabolism molecule *succinate receptor 1 (SUCNR1)* (Chen et al., 2023). In the context of engineered drug delivery, MSC-Exos can serve as low-immunogenicity nanocarriers to achieve targeted delivery of chemotherapeutic agents, thereby overcoming drug resistance and off-target effects associated with conventional chemotherapy. One study successfully loaded doxorubicin and the topoisomerase I inhibitor SN38 into MSC-Exos, enhancing their CRC cell targeting through surface modification. This approach significantly improved cytotoxicity while reducing systemic side effects, validating their potential as an intelligent drug delivery system (Bagheri et al., 2020; Pishavar et al., 2023). Despite these advances, clinical translation faces several hurdles. Storage stability remains a concern—even at −80 °C, prolonged storage can alter exosome morphology, surface properties, and protein content. Biodistribution studies showed that aptamer-decorated exosomes achieve higher tumor accumulation and faster liver clearance than non-targeted formulations. However, systematic evaluation of pharmacokinetics, immunogenicity, and immune clearance mechanisms is still needed. Future efforts must also address scalable GMP manufacturing, standardized dosing, and regulatory pathways to enable clinical application of these engineered exosome-based therapies. Furthermore, MSC-Exos play an important role in modulating the tumor immune microenvironment. Recent research demonstrates that murine B-MSC-Exos can serve as delivery vehicles for miR-125b-5p, attenuating the immunosuppressive function of regulatory T cells by downregulating tumor necrosis factor receptor 2 (TNFR2) and forkhead box protein P3 (Foxp3) expression. This remodeling of the tumor immune microenvironment enhances the efficacy of anti-programmed death receptor 1 (PD-1) immunotherapy in CRC treatment (Jiang M. et al., 2025).

**TABLE 1:** The effects of MSC-Exos on CRC.

Source of exosomes	Exosome type	*In Vitro*/*In Vivo*	Model system	Primary mechanism	Biological functions	References
B-MSC-Exos	Native	*In vitro* only	HCT116, SW480 cell lines	Downregulates mTOR signaling through miR-100 and miR-143	Inhibits tumor progression	Jahangiri et al. (2022)
B-MSC-Exos	Native and engineered (miR-431–5p overexpression)	*In vitro* and *in vivo*	LoVo cell line; BALB/c nude mouse xenograft model	Inhibits PRDX1 expression via miR-431–5p	Inhibits tumor progression and Promotes apoptosis	Qu et al. (2022)
B-MSC-Exos	Native and engineered (SCARA5 knockdown)	*In vitro* and *in vivo*	HCT116, LOVO, HT-29, SW480 cell lines; BALB/c nude mouse xenograft model	Inactivates PI3K/Akt through SCARA5	Inhibits tumor progression	Fang et al. (2023)
AD-MSC-Exos	Native	*In vitro* only	HCT116 cell line	Inhibits the expression of aquaporin-5 and epidermal growth factor receptor genes	Inhibits tumor progression and Promotes apoptosis	Mansourabadi et al. (2022)

#### 5.1.2 The pro-cancer effect of MSC-Exos on CRC

Studies have also demonstrated that MSC-Exos can promote CRC progression under specific conditions. This pro-tumor effect is primarily achieved through two mechanisms: direct regulation of malignant tumor cell behavior and remodeling of the immune microenvironment. With respect to direct modulation of malignant phenotypes, B-MSC-Exos have been shown to induce EMT, promote cancer cell proliferation, migration, and invasion, inhibit apoptosis, and thereby accelerate tumor growth and lung metastasis in colorectal cancer (Jing et al., 2025). Furthermore, under the influence of colon cancer cell-derived exosomes, AD-MSC-Exos can undergo transformation into cancer-associated fibroblasts. Through the signals they carry, these transformed exosomes activate transient receptor potential canonical channel 3 (TRPC3), which subsequently induces NF-κB signaling pathway activation. This signaling cascade drives the conversion of cancer-associated fibroblasts toward a mesenchymal phenotype, accelerating CRC growth, migration, and invasion. Notably, inhibition of exosome-mediated signaling significantly reduces NF-κB phosphorylation levels and reverses the malignant tumor phenotype, suggesting that TRPC3 may serve as a potential molecular target for CRC treatment (Xue et al., 2022). Developing inhibitors targeting the molecules in the above pathways to reverse the exosome-mediated pro-cancer effects provides an innovative direction for CRC prognosis.

### 5.2 Effect of MSC-Exos on liver cancer

#### 5.2.1 The anti-tumor effect of MSC-Exos on liver cancer

Liver cancer is the third most common cause of cancer-related deaths globally. In recent years, MSC-Exos have demonstrated multi-mechanistic intervention potential in liver cancer therapy due to their natural targeting ability, low immunogenicity, and cargo programmability (Table 2). MSC-Exos can directly regulate the malignant phenotype of liver cancer cells and their stem cells by delivering functional non-coding RNAs. For example, B-MSC-Exos can deliver the long non-coding RNA *C5orf66-AS1*, which acts as a competitive endogenous RNA, adsorbing miR-127–3p to upregulate the expression of dual specificity phosphatase 1 (DUSP1), inhibiting phosphorylation of the extracellular signal-regulated kinase pathway, thereby effectively suppressing malignant biological behaviors such as self-renewal, proliferation, migration, and invasion in hepatocellular carcinoma (HCC) (Gu et al., 2021). Similarly, U-MSC-Exos can inhibit HCC cell migration, invasion, and EMT by overexpressing miR-451a and reducing A Disintegrin And Metalloproteinase 10 (ADAM10), enhance cell apoptosis, and reverse paclitaxel resistance in HCC cells (Xu et al., 2021). In addition to directly delivering RNA, MSC-Exos can also act as a synergistic enhancer of chemical drugs. Studies have shown that U-MSC-Exos, when combined with the natural polyphenol compound gallic acid (GA), can exert significant synergistic anti-tumor effects through multi-pathway integration of metabolic and inflammatory signals. This combined strategy can simultaneously downregulate key glycolytic enzymes, pro-inflammatory factors, and affect core metabolic pathways such as selenium compound metabolism and cysteine-methionine metabolism, thereby systematically remodeling the tumor metabolic microenvironment, inhibiting cell proliferation and inducing apoptosis (Zhang P. et al., 2025). Collectively, these studies elucidate the molecular basis by which MSC-Exos exert anti-tumor effects through multi-target intervention in key liver cancer pathways and epigenetic regulation, providing promising new therapeutic targets for liver cancer treatment.

**TABLE 2:** The effects of MSC-Exos on HCC.

Source of exosomes	Exosome type	*In Vitro*/*In Vivo*	Model system	Primary mechanism	Biological functions	References
B-MSC-Exos	Engineered (miR-338–3p mimic transfection)	*In vitro* only	HCC cell line HepG2	Downregulates EST1 via miR-338–3p	Inhibits tumor progression and Promotes apoptosis	Li Y. H. et al. (2021)
U-MSC-Exos	Engineered (overexpression/knockdown of lncRNA FAM99B via plasmid/siRNA transfection)	*In vitro* and *in vivo*	Human HCC cell lines: MHCC97L, MHCC97H, HepG2, Hep3B; Normal: LO2	lncRNA FAM99B overexpression inhibits cell cycle	Inhibits tumor progression and Promotes apoptosis	Xu et al. (2024)
U-MSC-Exos	Engineered (miR-27a-3p mimics/inhibitor transfection)	*In vitro* and *in vivo*	Human HCC cell lines: Huh7, HepG2, MHCC97H, Hep3B; Normal: L02	Downregulates Golgi membrane protein 1 expression via miR-27a-3p	Inhibits tumor progression	Bongolo et al. (2022)

#### 5.2.2 The pro-tumor effect of MSC-Exos on liver cancer

MSC-Exos exert a dual role in liver cancer progression. Under specific microenvironmental conditions, particularly hypoxia, the molecular composition of MSC-Exos may undergo reprogramming, shifting their function from tumor suppression to tumor promotion. One study demonstrated that under hypoxic conditions, the expression of miR-652–3p in B-MSC-Exos is significantly upregulated and directly transferred to liver cancer cells. There, it targets and inhibits the expression of trinucleotide repeat containing gene 6A (TNRC6A), thereby attenuating its tumor-suppressive function and promoting cancer cell proliferation and metastasis (Li Z. et al., 2023). Notably, hypoxia is often accompanied by oxidative stress, which is one of the core characteristics of the tumor microenvironment (Xing et al., 2022). Reactive oxygen species (ROS) not only directly modulate tumor cell behavior but also reshape the microenvironment by influencing exosomal miRNA packaging and delivery (Park et al., 2025). Moreover, ROS-mediated signaling pathways play a critical role in maintaining cancer stem cell stemness; therefore, targeting ROS-induced cancer stemness has emerged as a promising therapeutic strategy (Du et al., 2024).

### 5.3 Effect of MSC-Exos on gastric cancer

#### 5.3.1 The anti-tumor effect of MSC-Exos on gastric cancer

Gastric cancer (GC), a common malignancy of the digestive system, is associated with a low 5-year survival rate, underscoring the urgent need for novel therapeutic strategies to improve patient prognosis. MSC-Exos have demonstrated substantial potential in GC treatment owing to their precise targeting and delivery capabilities (Table 3). In the context of therapeutic molecule delivery, MSC-Exos can serve as safe and efficient carriers. For instance, B-MSC-Exos delivering the coding gene of the hepatocyte growth factor antagonist NK4 enable sustained release of the NK4 protein at tumor sites, effectively inhibiting angiogenesis and inducing apoptosis while avoiding the immunogenic risks associated with traditional viral vectors (Zhu et al., 2014). Additionally, engineered B-MSC-Exos can function as chemotherapy drug delivery systems. B-MSC-Exos loaded with doxorubicin, in combination with small interfering RNA targeting the long non-coding RNA PVT1, synergistically inhibit the proliferation, migration, and invasion of gastric cancer cells, while significantly suppressing tumor growth and metastasis in xenograft models (Ouyang et al., 2025). Moreover, the endogenous microRNA-1228 in B-MSC-Exos can inhibit the proliferation, invasion, and migration of GC cells by targeting *matrix metalloproteinase-14* (Chang et al., 2021). Notably, the functionality of MSC-Exos is plastic and adaptable to the microenvironment. Studies have found that co-culturing GC cells with human bone marrow mesenchymal stem cells promotes the secretion of pro-cancer exosomes. After pre-treatment with oxaliplatin, miR-424–3p expression in B-MSC-Exos is significantly upregulated, which inhibits EMT by suppressing the Rhox homeobox family member F2, thereby inhibiting tumor progression (Shen et al., 2024). This finding reveals that chemotherapy drugs can reshape exosome function, converting them from pro-cancer mediators to tumor-suppressive carriers, forming a therapy-triggered functional reversal mechanism. In summary, the concept of drug-triggered functional reversal of exosomes offers a new perspective for gastric cancer treatment. Future therapeutic strategies may not be limited to directly killing tumor cells but could also involve regulating exosome signaling within the tumor microenvironment, shifting their function from supporting the tumor to inhibiting it, thereby advancing the treatment paradigm from pure cytotoxicity to microenvironment remodeling.

**TABLE 3:** The effects of MSC-Exos on GC.

Source of exosomes	Exosome type	*In Vitro*/*In Vivo*	Model system	Primary mechanism	Biological functions	References
B-MSC-Exos	Native	*In vitro* and *in vivo*	Mouse BMMSCs (p53+/+ and p53−/−); Mouse gastric cancer MFC cells	Regulate the Wnt/β-catenin signaling pathway	Promotes tumor progression	Mao et al. (2017)
U-MSC-Exos	Native	*In vivo* only	Gastric cancer cell line HGC-27	Activate the Akt pathway and promote EMT	Promotes tumor progression	Gu et al. (2016)

#### 5.3.2 The pro-tumor effect of MSC-Exos on gastric cancer

In recent years, the mechanisms by which MSC-Exos regulate the malignant phenotype of gastric cancer through multiple pathways have gradually been elucidated. Studies have found that B-MSC-Exos can directly enhance the proliferative capacity of gastric cancer cells by transferring miR-221, promoting cell migration, invasion, and matrix adhesion properties (Ma et al., 2017).

### 5.4 Effect of MSC-Exos on pancreatic cancer

Pancreatic cancer (PC) has a poor prognosis and is one of the most lethal malignant tumors globally, with an extremely high fatality rate. Current treatment methods for PC remain limited, and its prognosis continues to be poor. Studies suggest that exosomes hold promise as a new strategy for treating PC (Table 4). MSC-Exos can regulate the biological behavior of PC by delivering functional molecules. For example, B-MSC-Exos can deliver the miR-143–3p they carry to PC cancer cells, and by targeting long non-coding RNAs, inhibit key carcinogenic signaling pathways, ultimately suppressing PC cell proliferation, migration, and invasion, and promoting cell apoptosis (Wang et al., 2021). In terms of immune regulation, *circ_0006790* carried by B-MSC-Exos has been found to remodel the tumor immune microenvironment through epigenetic modifications. Specifically, *circ_0006790* promotes the nuclear translocation of chromobox protein 7 by binding to it, catalyzing hypermethylation of the promoter region of the tumor suppressor gene *S100 calcium-binding protein A11*, thereby inhibiting its transcriptional activity. This process significantly reduces the expression levels of PD-L1 and cytotoxic T-lymphocyte-associated antigen 4 (CTLA-4) in cancer cells, ultimately enhancing the infiltration and killing function of CD8^+^ T cells, reversing tumor immune escape (Gao et al., 2022). Additionally, engineered exosomes offer a new approach for targeted therapy. The use of *yCD::UPRT* suicide gene-engineered exosomes derived from pancreatic cancer-associated fibroblasts and mesenchymal stem cells from different tissues can efficiently target pancreatic ductal adenocarcinoma (PDAC) cells and their fibrotic microenvironment. These exosomes convert the prodrug 5-fluorocytosine into 5-fluorouracil inside the cell, generating a strong synergistic anti-tumor effect *in vitro* and in tumor microenvironment models, and demonstrating good safety in mice, providing a new method for targeted cell therapy for cancer. Despite these promising preclinical findings, future efforts should prioritize optimizing isolation techniques, establishing standardized quality control protocols, and conducting systematic evaluations of long-term stability, safety, and *in vivo* efficacy to facilitate clinical translation of this combination therapeutic approach (Jakubechova et al., 2025).

**TABLE 4:** The effects of MSC-Exos on digestive system tumors.

Cancer	Source of exosomes	Exosome type	*In Vitro*/*In Vivo*	Model system	Primary mechanism	Biological functions	References
PC	U-MSC-Exos	Engineered (miR-128–3p mimic/inhibitor transfection)	*In vitro* only	PDAC cell lines: PANC-1, BxPC-3, Capan-2, CFPAC-1; Normal: hTERT-HPNE	hsa-miR-128–3p targets Galectin-3	Inhibits tumor progression	Xie et al. (2022)
CCA	U-MSC-Exos	Engineered (miR-15a-5p mimic/inhibitor transfection)	*In vitro* and *in vivo*	CCA cell lines: HuCCT1, HuH28; HEK-293T	miR-15a-5p targets and inhibits CHEK1 expression	Inhibits tumor progression and promotes apoptosis	Li and Wang (2022)
	U-MSC-Exos	Engineered (circ_0037104 overexpression via plasmid transfection)	*In vitro* and *in vivo*	CCA cell lines: TFK-1, HuCCT1	Suppression of miR-620 and derepression of AFAP1	Inhibits tumor progression	Yuan et al. (2024)

Standard chemotherapy regimens for PC have long been hindered by acquired resistance to gemcitabine. B-MSC-Exos, as natural nanoparticle carriers, can overcome this bottleneck by enhancing tumor targeting and modulating drug resistance-related pathways. Experiments have confirmed that B-MSC-Exos loaded with gemcitabine exhibit potent cytotoxicity against PC cells, promoting cell apoptosis and inhibiting tumor progression (Tang et al., 2024). To further enhance therapeutic effects, research has progressed from single-drug delivery to multi-drug co-delivery and combination therapy. When B-MSC-Exos co-load paclitaxel and the active metabolite of gemcitabine, gemcitabine monophosphate, a dual-drug co-delivery system can be constructed, which exhibits significant synergistic anti-tumor effects both *in vitro* and *in vivo*, effectively inhibiting tumor growth, inducing cell apoptosis, and activating relevant immune responses (Zhou et al., 2020). Furthermore, engineering a B-MSC-Exos dual-functional system co-loaded with oxaliplatin and galectin-9 small interfering RNA (galectin-9 siRNA) can simultaneously induce immunogenic cell death and reverse the immunosuppressive microenvironment, significantly enhancing the immunotherapeutic effect in pancreatic cancer (Zhou et al., 2021).

On the other hand, some studies suggest that exosomes may promote PC progression. Ding et al. (Ding et al., 2021) found that U-MSC-Exos deliver tumor-associated miRNAs such as miR-100–5p, activating pathways related to cell glucuronidation, ascorbate metabolism, and aldose metabolism, significantly promoting the proliferation, invasion, and growth of PDAC.

### 5.5 The effects of MSC-Exos on other gastrointestinal tumors

Esophageal cancer (EC) is a common and lethal malignant tumor worldwide. In recent years, research on exosomes in the field of EC has advanced, revealing that the expression of *Enabled homolog (ENAH)* is positively correlated with the malignant phenotype of esophageal squamous cell carcinoma. The miR-375 delivered by U-MSC-Exos can target and inhibit *ENAH* expression, thereby suppressing the proliferation, invasion, migration, while promoting apoptosis of esophageal cancer cells (He et al., 2020).

Cholangiocarcinoma (CCA) is a malignant tumor that originates from bile duct epithelial cells. MSC-Exos play an important role in CCA treatments (Table 4). In terms of drug delivery and chemotherapy sensitization, MSC-Exos can serve as efficient nanocarriers, improving the efficacy and safety of conventional chemotherapy drugs. B-MSC-Exos, as an efficient drug carrier, can effectively load and target the delivery of 5-fluorouracil, significantly improving its anti-tumor effect and inhibiting the proliferation of CCA cells. Due to their good biocompatibility and low toxicity, exosomes have significant potential in CCA treatment (Chen et al., 2022). In anti-angiogenesis and immune microenvironment regulation, engineering modifications have further expanded the functions of MSC-Exos. Researchers have constructed a B-MSC-Exos delivery system based on chitosan (CS) hydrogel, which utilizes the positively charged characteristics of CS to stabilize the binding with the negatively charged exosome membrane through electrostatic interactions, forming an efficient encapsulation system. This delivery system not only significantly enhances the *in vivo* stability and retention time of exosomes, but also enables the slow, sustained release of Tissue Inhibitor of Metalloproteinase 2 (*TIMP2*), effectively inhibiting the activity of Vascular Endothelial Growth Factor A and its receptor (VEGFA/VEGFR2), blocking tumor angiogenesis. At the same time, the system inhibits CCA cell proliferation and survival by downregulating the Wnt/β-catenin signaling pathway. Bioinformatics analysis further indicates that high expression of TIMP2 is associated with increased immune cell infiltration in the tumor microenvironment, suggesting that this strategy may synergistically regulate the immune microenvironment while inhibiting angiogenesis. In conclusion, the chitosan hydrogel-based delivery system, with its stable electrostatic loading and sustained release characteristics, provides a new therapeutic strategy with continuous anti-angiogenesis and potential immune modulation functions for stromal-rich, highly vascularized cholangiocarcinoma (Song et al., 2025).

**FIGURE 2:**
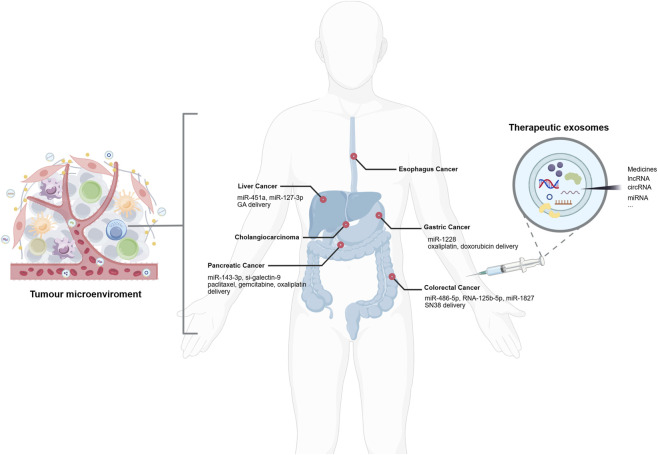
The effects of MSC-Exos on digestive system tumors. Created with BIOGDP.com (Jiang S. et al., 2025).

In summary, MSC-Exos play an important role in gastrointestinal tumors (Figure 2). The dual tumor-promoting and tumor-suppressive effects of MSC-Exos result from the coordinated interplay of multiple factors. First, the tissue source of MSCs determines the inherent functional propensity of their exosomes. Exosomes derived from bone marrow, umbilical cord, and adipose tissue exhibit distinct tumor-suppressive profiles due to differences in their molecular cargo. Second, microenvironmental stresses can reprogram exosomal functions. Hypoxia-induced enrichment of miR-210–3p and miR-652–3p promotes tumor progression, while exposure to chemotherapeutic agents may reverse the functional direction of exosomes. Third, engineering strategies can specifically enhance antitumor effects. Approaches such as drug loading, genetic modification, and siRNA co-delivery have enabled the construction of exosomes with controllable functions. Fourth, the recipient tumor type influences functional outcomes, with the same exosome exerting opposing effects in different tumors. Thus, MSC-Exo function is a consequence of the interplay between source properties, microenvironmental signals, engineering interventions, and tumor characteristics. Future research should shift from descriptive observations to mechanistic dissection, and develop functionally controllable engineered exosomes based on key regulatory nodes.

## 6 Conclusion and future perspectives

This review summarizes the complex roles of MSC-derived exosomes in gastrointestinal tumors. These vesicles can either suppress or promote cancer progression depending on their cellular origin, cargo, and tumor microenvironment. While many studies report tumor-suppressive effects through delivery of specific microRNAs or proteins, pro-tumorigenic activities have also been well documented, including enhanced proliferation, angiogenesis, and chemoresistance. The dual nature of MSC-Exos presents both opportunities and challenges for clinical translation. On one hand, their intrinsic cargo-loading capacity and intercellular communication functions make them attractive therapeutic vectors. On the other hand, the risk of unintended pro-tumorigenic effects necessitates rigorous safety evaluations prior to clinical application.

Notably, several limitations and research gaps in the current field should be acknowledged: significant heterogeneity exists across studies in terms of MSC sources, culture conditions, isolation methods, and exosome characterization, making direct comparisons challenging; the regulatory mechanisms governing exosomal cargo sorting and packaging, particularly under dynamic microenvironmental conditions, remain incompletely elucidated; most studies are still at the preclinical stage, lacking standardized production protocols and quality control metrics; furthermore, data on long-term *in vivo* biodistribution, biodegradation, and potential immunogenicity of MSC-Exos remain limited. These research gaps warrant further investigation to advance clinical translation of this field.

Future research should address several key directions. First, mechanistic studies are needed to decipher how specific cargo molecules and sorting pathways determine the net biological output. Second, integrating computational and systems biology approaches—including multi-omics data analysis, network modeling, and artificial intelligence—will help decode exosome heterogeneity and predict context-dependent functions across different tumor types and microenvironments. Such integrative strategies have been successfully applied in stem cell research to resolve transcriptional heterogeneity, reconstruct gene regulatory networks, and identify key regulators of cell fate decisions using single-cell sequencing and machine learning frameworks (Raghav et al., 2025). These computational methodologies are readily adaptable to MSC-Exos research, enabling systematic dissection of their molecular complexity and functional diversity. Third, engineering strategies should be refined to develop exosomes that deliver therapeutic cargo while minimizing potential risks, possibly through targeted modification of surface ligands or cargo composition.

Collectively, a deeper understanding of MSC-Exos biology, combined with advanced computational and experimental tools, will guide more rational design of exosome-based therapies and pave the way toward safe and effective clinical applications.
